# The Octyl Ester of Ginsenoside Rh2 Induces Lysosomal Membrane Permeabilization via Bax Translocation

**DOI:** 10.3390/nu8050244

**Published:** 2016-04-25

**Authors:** Fang Chen, Bing Zhang, Yong Sun, Zeng-Xing Xiong, Han Peng, Ze-Yuan Deng, Jiang-Ning Hu

**Affiliations:** 1 State Key Laboratory of Food Science and Technology, Institute for Advanced Study, Nanchang University, Nanchang, Jiangxi 330047, China; xinganchenfang@163.com (F.C.); zhangbingair@hotmail.com (B.Z.); yong.sun@canada.ca (Y.S.); m15179146869@163.com (Z.-X.X.); ncuskpenghan@163.com (H.P.); dengzy@ncu.edu.cn (Z.-Y.D.); 2 College of Food Science, Nanchang University, Nanchang, Jiangxi 330047, China

**Keywords:** Ginsenoside Rh2, octyl ester derivative, apoptosis, lysosomal membrane permeabilization, mitochondria dysfunction, Bax

## Abstract

Ginsenoside Rh2 is a potential pharmacologically active metabolite of ginseng. Previously, we have reported that an octyl ester derivative of ginsenoside Rh2 (Rh2-O), has been confirmed to possess higher bioavailability and anticancer effect than Rh2 *in vitro*. In order to better assess the possibility that Rh2-O could be used as an anticancer compound, the underlying mechanism was investigated in this study. The present results revealed that lysosomal destabilization was involved in the early stage of cell apoptosis in HepG2 cells induced by Rh2-O. Rh2-O could induce an early lysosomal membrane permeabilization with the release of lysosomal protease cathepsins to the cytosol in HepG2 cells. The Cat B inhibitor (leu) and Cat D inhibitor (pepA) inhibited Rh2-O-induced HepG2 apoptosis as well as tBid production and Δφm depolarization, indicating that lysosomal permeabilization occurred upstream of mitochondrial dysfunction. In addition, Rh2-O induced a significant increase in the protein levels of DRAM1 and Bax (*p* < 0.05) in lysosomes of HepG2 cells. Knockdown of Bax partially inhibited Rh2-O-induced Cat D release from lysosomes. Thus it was concluded that Rh2-O induced apoptosis of HepG2 cells through activation of the lysosomal-mitochondrial apoptotic pathway involving the translocation of Bax to the lysosome.

## 1. Introduction

Ginsenoside Rh2, one of the major effective metabolites of ginseng, has been reported to suppress growth in various cancer cell lines [1,2,3]. However, Rh2 has limited application in medical supplies due to its low oral bioavailability as well as high toxicity to normal cells [4,5]. Rh2-O, a novel mono-octanoyl ester derivative of Rh2, was then designed in our lab aiming at significantly enhancing the oral bioavailability of Rh2 [6]. Caco-2 cell monolayers have been generally accepted as an *in vitro* model for prediction of drug absorption across the human intestine due to their morphologic and functional similarities to human small intestinal epithelial cells [7]. Our previous study by Zhang *et al.* (2014) found that Rh2-O possessed a better absorption than Rh2 in the Caco-2 system, and the transport mechanisms for both Rh2 and Rh2-O were transcellular passive diffusion [8]. Chen *et al.* reported that the IC_50_ value of Rh2-O for inhibition of HepG2 cell proliferation was 20.15 μM, which was approximately half the amount of the IC_50_ value of Rh2 [6]. Meanwhile, the findings suggested that Rh2-O induced caspase-dependent apoptosis via the intrinsic pathway. These studies have confirmed that Rh2-O may be more efficient than Rh2 in anticancer activity. In order to better assess the possibility that Rh2-O could be used as an anti-cancer compound, the related mechanism needs to be further elucidated.

Interestingly, some investigators suggested that the proteins of the Bcl-2 family that mediate mitochondrial membrane permeabilization might also be involved in lysosomal membrane permeabilization. Lysosomes are usually considered to play an important role in autophagy to provide digestive enzymes. During recent years, it has been reported that the lysosomes have been implicated in the regulation of cell apoptosis [9,10]. It is well known that Bax is central to the regulation of mitochondrial membrane permeabilization and its action is counteracted by Bcl-2 [11]. Bax has, however, also been reported to be involved in lysosomal membrane permeabilization when incubated with pure lysosomal fractions [12]. Guan and colleagues recently found that the interaction between Bax and DRAM1 could result in the insertion of Bax to the lysosomal membrane and the release of Cat B [13]. Lysosomal membrane permeabilization and the release of enzymes from the lysosomes to the cytosol followed by cell apoptosis have been reported [14,15]. It was found that lysosomal membrane permeabilization was initiated in the early phase of apoptosis by lysosomotropic detergents, serum withdrawal, oxidative stress or tumor necrosis factor-α and subsequently released lysosomal cathepsins [16,17,18,19]. The lysosomal protease cathepsins have been recognized as potent inducers of programmed cell death. The early release of lysosomal enzymes may cause mitochondrial damage, followed by cytochrome c release, apoptosome formation with Apaf-1, and caspase activation. For example, the released cathepsins could activate Bid to form a truncated BH3-interacting domain death agonist (tBid) [20]. tBid relocates to the mitochondria and may trigger mitochondrial membrane permeabilization and the release of cytochrome *c* (Cyt C) [21].

The aim of this study was to determine whether lysosomal membrane permeabilization is involved in Rh2-O-induced HepG2 cell apoptosis, or if the release of cathepsins as the upstream signaling process could lead to mitochondrial dysfunction. In addition, we investigated how DRAM1 and Bax mediated lysosomal membrane permeabilization. The present study has provided novel information for understanding the molecular mechanisms by which Rh2-O induced apoptosis in HepG2 cells.

## 2. Experimental Section

### 2.1. Chemicals and Antibodies

Rh2-O was synthesized in our laboratory. Normal growth media (MEM) and fetal bovine serum (FBS) were purchased from Gibco-BRL Co. (Grand Island, NY, USA). 3-(4,5-dimethylthiazole-2-yl)-2,5-diphenyltetrazolium bromide (MTT), proteinase K, 2′,7′-dichlorofluorescin diacetate (DCFH-DA), phenylmethanesulfonyl fluoride (PMSF) and leupeptin (Leu) were purchased from Sigma Chemical Co. (St. Louis, MO, USA). AnnexinV-FITC apoptosis detection kit was from B.D. Clontech Laboratories (Mountain View, CA, USA). Rabbit anti-human antibodies to Cat B, cathepsin D (Cat D), tBid, Bax and DRAM1 were from Santa Cruz Biotechnology Co. (Santa Cruz, CA, USA). Antibodies against β-actin, anti-mouse and anti-rabbit IgG-HRP were purchased from TransGen Biotechnology Co. (Beijing, China). All other compounds had a purity of ≥98%.

### 2.2. Cell Culture and Treatment

Human hepatoma HepG2 cells were procured from the National Centre for Cell Sciences (NCCS), China. HepG2 cells were maintained in MEM medium containing 10% FBS, 100 units/mL penicillin and 100 μg/mL streptomycin. Cells were grown in an incubator at 37 °C with 95% humidity and 5% CO_2_. Cells were treated with Rh2-O (dissolved in DMSO), while the untreated cultures received only the vehicle (DMSO < 0.2%).

### 2.3. Lysosomal Stability Assessments

The induction of lysosomal membrane permeabilization by the Rh2-O was analyzed using the acridine orange (AO) relocation method [22]. AO is a metachromatic fluorophore. Oligomeric form and protonated AO (AOH^+^), at high concentrations in intact lysosomes, exhibited red fluorescence. The monomeric deprotonated form of AO, at low concentrations in nuclear and cytosolic, exhibited green fluorescence. HepG2 cells were seeded to a six-well plate for 16 h and then exposed to 17.5 μM of Rh2-O for 24 h. After incubation, cells were stained with AO (5 μM, 30 min, 37 °C) to label lysosomes and then examined with a fluorescence Olympus BX50 microscope with the corresponding filter, 470–490 nm excitation and 515 nm emission wavelengths. 

### 2.4. Assay of Cell Proliferation by MTT

Cells were seeded onto 96-well plates for 16 h, and then pretreated with Leu (0, 0.5, 1.0, 2.0 and 4.0 μM) or pepA (0, 10, 20, 40 and 60 μM) for 1 h before being exposed to 17.5 μM of Rh2-O in the presence of Leu or pepA for 24 h in a final volume of 180 μL/well. Then, 20 μL of MTT (10 mM stock solution of saline) was added to each well and culture was continued at 37 °C for 4 h. After incubation, the supernatant was discarded while the MTT-formazon crystals were dissolved in 150 μL of DMSO for each well. The optical density (OD) was measured at 490 nm with reference wavelength of 620 nm. Cell growth, as the percent viability, was calculated by comparing the absorbance of treated *vs.* untreated cells.

### 2.5. Flow Cytometric Analysis of Apoptosis 

The apoptosis cells were determined by double-staining the HepG2 cells with AnnexinV-FITC and PI through flow cytometry. HepG2 cells were seeded onto a six-well plate for 16 h and then pretreated with Leu (10 μM) or pepA (1.0 μM) for 1 h before exposure to 17.5 μM of Rh2-O in the presence of Leu or pepA for 24 h. After incubation, cells were collected, washed twice with ice-cold PBS and stained with AnnexinV-FITC and PI as per manufacturer’s instructions (BD Biosciences, PHarmingen). Cells were scanned in FL-1 (FITC) *versus* FL-3 (PI) channels on BD-LSR flow cytometer, using quadrant statistics for apoptotic cell populations. At least 10,000 cells were analyzed for each sample. 

### 2.6. Measurement of Mitochondrial Membrane Potential (ΔΨmt)

ΔΨmt was measured by using mitochondrial tracking fluorescent JC-1. HepG2 cells were seeded onto a six-well plate for 16 h and then pretreated with pepA (1.0 μM) for 1 h before being exposed to 17.5 μM of Rh2-O in the presence of pepA for 24 h. After incubation, cells were washed and stained with 10 μg/mL of JC-1 at 37 °C for 10 min. Cells were then washed with PBS and the decrease in green fluorescence was analyzed with a fluorescence microscope (Olympus Optical Co., Tokyo, Japan). The green fluorescence was observed under blue light, and the red fluorescence was observed under green light.

### 2.7. Culture Conditions to Inhibit Gene Expression by siRNA.

In HepG2 cells, siRNA was applied to inhibit Bax expression using siRNA in a lipotransfection assay. Exponentially growing cells were suspended in the 1.25 mL of transfection medium devoid of growth media and antibiotics. The cells received the siRNA solution and the transfection reagent, mixed along with transfection medium, and the plate was incubated for 6 h according to the instructions provided by the supplier (Santa Cruz Biotechnology, Santa Cruz, CA, USA). At the end of the transfection, cells were incubated with 1.5 mL of MEM containing 2 × FBS. After 18–24 h, the cells were treated with 17.5 μM of Rh2-O for another 24 h. The whole cell lysates were further prepared in the similar manner as described below.

### 2.8. Preparation of Whole Cell Lysates

HepG2 cells were seeded onto a six-well plate for 16 h and then pretreated with pepA (1.0 μM) for 1 h before being exposed to 17.5 μM of Rh2-O in the presence of pepA for 24 h. The cells were harvested and washed twice with ice-cold PBS and then lysed by 300 μL of RIPA lysate each well on ice. After centrifugation at 16,000× *g* for 15 min, supernatant was taken and stored at −80 °C until use.

### 2.9. Preparation of Lysosomal Lysates

The isolation of lysosomes was performed with Lysosome Enrichment Kit for Tissue and Culture Cells (Thermo Fisher Scientific Inc.; Waltham, MA, USA). HepG2 cells were plated to a culture dish (100 × 200 mm) for 16 h and then exposed to 17.5 μM of Rh2-O for 24 h. The cells were harvested and then homogenized. Homogenates were then centrifuged at 500× *g* at 4 °C for 10 min and the supernatants were collected. The supernatants were then mixed with OptiPrep Cell Separation Media and centrifuged at 145,000× *g* at 4 °C for 2 h. The lysosomal band was lysed with 2% CHAPS in PBS.

### 2.10. Protein Determination and Western Blot Analysis

The protein contents were determined using Bradford reagent (Bio-Rad protein assay kit, Bio-Rad Laboratories Co., Ltd., Shanghai, China) and aliquots normalized to equal quantities before loading. The lysates were resolved on SDS-PAGE analysis and electro-transferred to NC membranes (Bio-Rad Laboratories Co., Ltd., Shanghai, China) at 70 V, 4 °C for 2 h. The membranes were blocked in TBST buffer containing 5% skimmed milk for 2 h and blotted with respective rabbit anti-human primary antibodies overnight at 4 °C. Blots were washed in TBST and incubated with HRP-conjugated secondary antibody. Protein bands were detected using enhanced chemiluminescent reagent (ECL kit, Amersham Biosciences, Arlington Heights, IL, USA). The density of the bands was arbitrarily quantified using ImageJ64 software (NIH, Bethesda, MD, USA).

### 2.11. Statistical Analysis

Results were analyzed by Student’s *t*-test or one-way analysis of variation (ANOVA) with Duncan’s multiple range tests using SPSS 13.0 software (SPSS, Inc., Chicago, IL, USA). Data were presented as the mean ± standard error of the mean (SEM) of at least three separate experiments. Densitometric analysis of Western blot results was carried out by Image J64 software (NIH, Bethesda, MD, USA).

## 3. Results

### 3.1. Rh2-O Induced Lysosomal Membrane Permeabilization in HepG2 Cells

**Figure 1:**
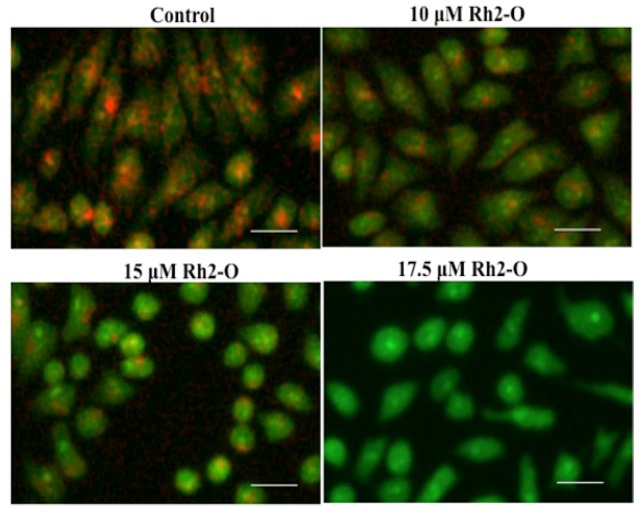
Lysosomal membrane permeabilization induced by Rh2-O treatment. Cells plated onto a six-well plate were incubated with or without Rh2-O. After Rh2-O treatment, lysosomes were stained with 5 μM of acridine orange and examined by fluorescence microscope. The scale bar is 50 μm.

AO is a lysosomotropic metachromatic fluorophore. AO exhibits a red fluorescence when it is accumulated within intact lysosomes and yields in green fluorescence after diffusion into the cytosol after lysosomal membrane permeabilization [23]. As shown in Figure 1, 24-hour treatment of HepG2 cells with Rh2-O led to a dose-dependent decrease in the red fluorescence and increase in the green fluorescence. The results provided first evidence that Rh2-O-treated HepG2 cells underwent lysosomal membrane permeabilization, suggesting that lysosomal destabilization occurred in the apoptosis cascade induced by Rh2-O.

### 3.2. Rh2-O Triggered the Release of Cathepsin to the Cytosol

**Figure 2:**
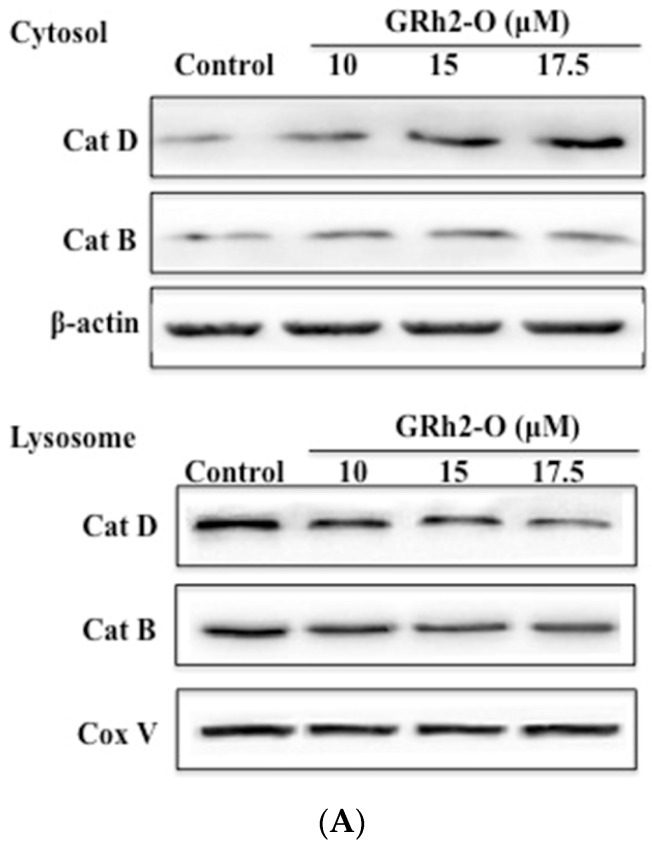

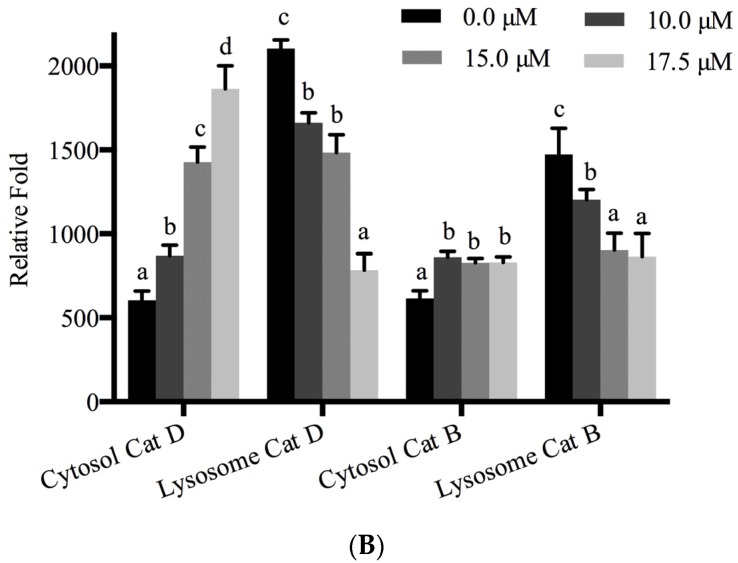
The levels of Cat D (cathepsin D) and Cat B (cathepsin B) in cytosol and lysosome in Rh2-O-treated HepG2 cells. (**A**) The protein levels of Cat D and Cat B in cytosolic and lysosomal fractions of Rh2-O-treated cells were determined by Western blot assay; (**B**) Histogram represents quantification of Cat D and Cat B protein expression levels in Rh2-O-stimulated HepG2 cell samples using Image J64 software (level of control cells/β-actin defined as 1). Results are presented as mean ± SD (standard deviation) of three separate experiments. Bars with different letters in each group are significantly different from each other (*p* < 0.05).

It was reported that lysosomal destabilization followed by the release of cathepsin proteases from lysosomes was a necessary step for the activation of downstream pathways leading to cell death [11,12]. In the present study, Western blot analysis was used to evaluate the release of Cat B and Cat D. Cells were treated with Rh2-O at different doses for 24 h and then cytosolic and lysosomal lysates were fractionated. The results showed that the levels of Cat B and Cat D were decreased in lysosomes and increased in cytosol after treatment with Rh2-O (Figure 2). The results showed that Rh2-O triggered the release of cathepsins to the cytosol in HepG2 cells. In addition, the results showed that the release of Cat D was more significant than Cat B (*p* < 0.05).

### 3.3. Effect of Cathepsin Inhibitors on Rh2-O-Induced Apoptosis in HepG2 Cells

**Figure 3:**
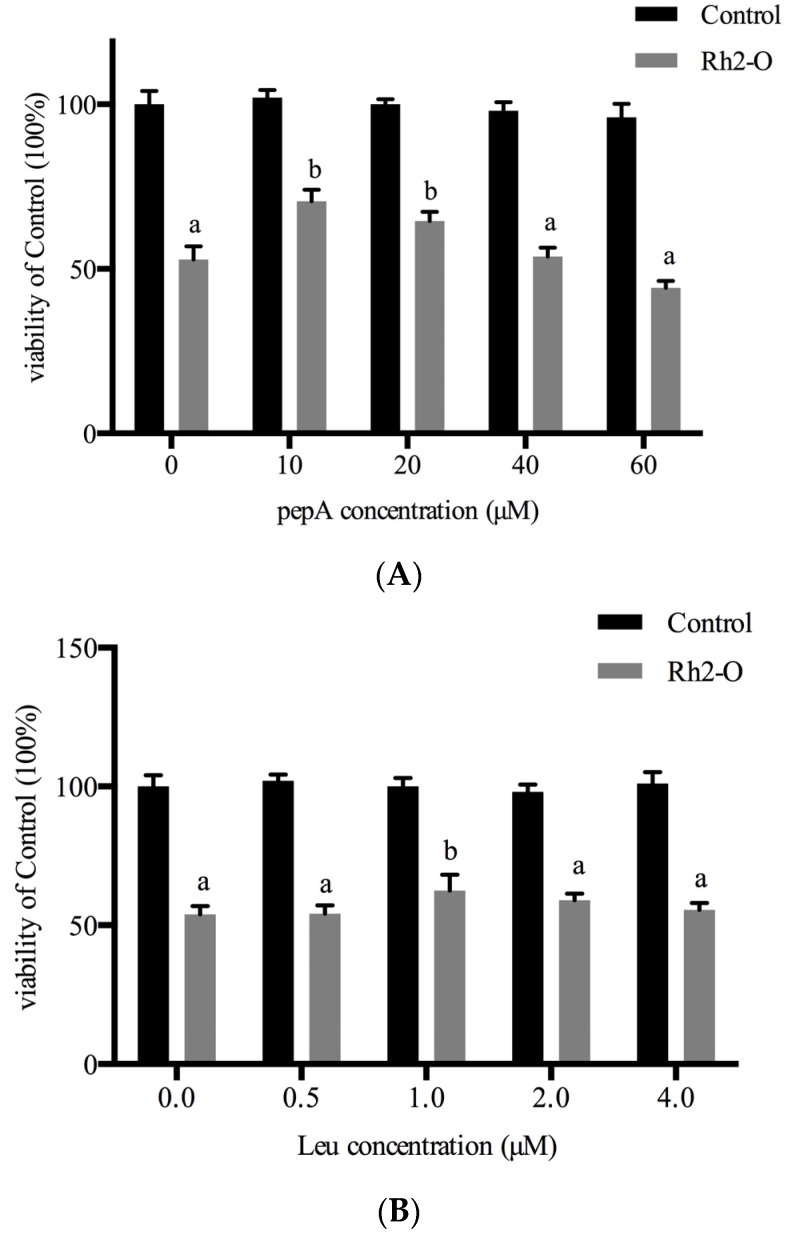

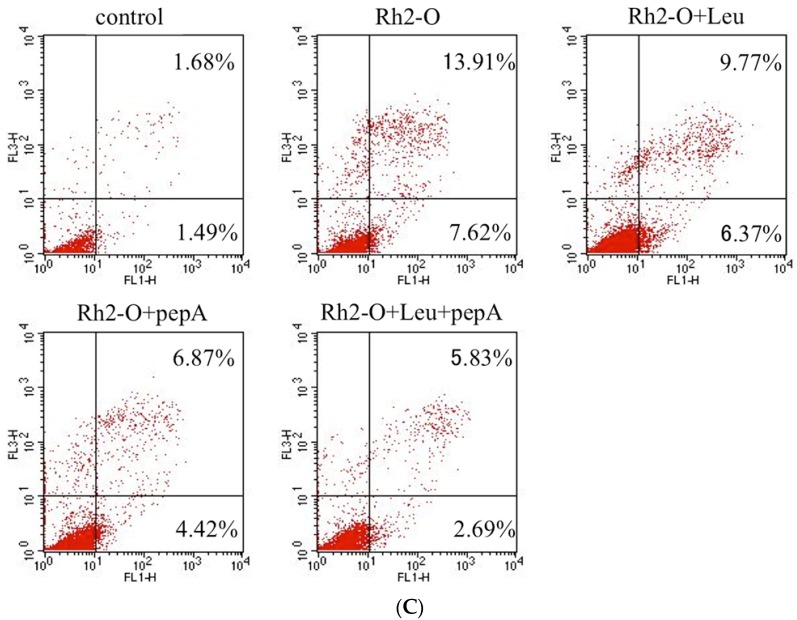
Effect of cathepsin inhibitors on Rh2-O-induced apoptosis in HepG2 cells. Cell growth was determined by the MTT method. HepG2 cells pretreated with pepA (the inhibitor of cathepsin D) (**A**) or Leu (**B**) (the inhibitor of cathepsin B) at different concentrations for 1 h and then incubated with or without 17.5 μM of Rh2-O. Results are presented as mean ± SD of three separate experiments (*n* = 5 per experiment). Bars with different letters are significantly different from each other (*p* < 0.05); (**C**) Flow cytometric analysis of Rh2-O induced apoptosis with or without pepA and Leu in HepG2 cells using Annexin V-FITC/PI. In upper quadrants: right, percentage of cells labeled with PI; left, percentage of cells labeled with PI and Annexin V. In lower quadrants: right, percentage of cells labeled with Annexin V; left, percentage of viable cells. Image data is a representative of one of three similar experiments.

To further determine the role of Cat B in Rh2-O-induced cell death, the cell viability and apoptosis were examined after pretreatment with Leu (Cat B inhibitor) or pepA (Cat D inhibitor). As shown in Figure 3A,B, pretreatment of HepG2 cells with increasing concentrations of inhibitors for 1 h before exposure to Rh2-O in the presence of inhibitors meant growth inhibitory activity induced by Rh2-O was decreased, albeit not completely. In addition, compared with the Rh2-O (17.5 μM)-treated group, the apoptotic populations in groups of Rh2-O (17.5 μM) with pepA (10 μM) group and Rh2-O (17.5 μM) with Leu (1 μM) were decreased by 11.29% and 16.14%, respectively (Figure 3C). A lower percentage of cell apoptosis (8.52%, corresponding to the effect of each individual inhibitor on cell apoptosis) was also observed when cells were treated with pepA and Leu. These results suggested that cathepsins might partially mediate Rh2-O cytotoxicity.

### 3.4. Cathepsins Caused the Cleavage of Bid

**Figure 4:**
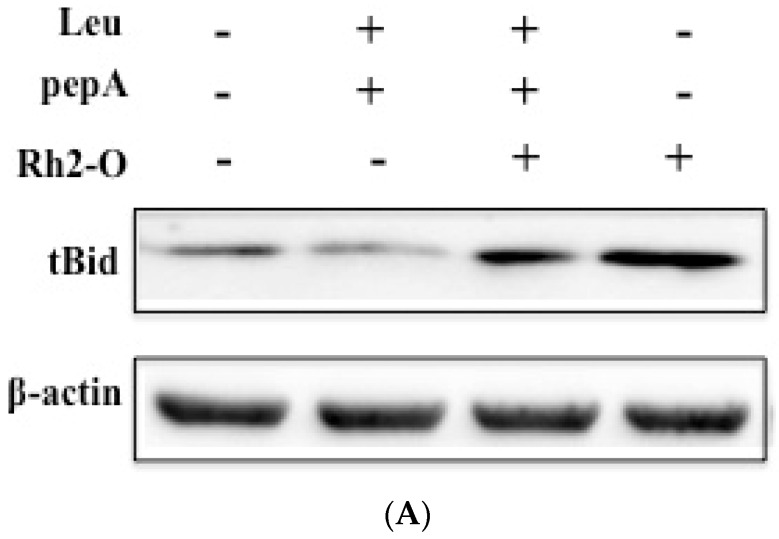

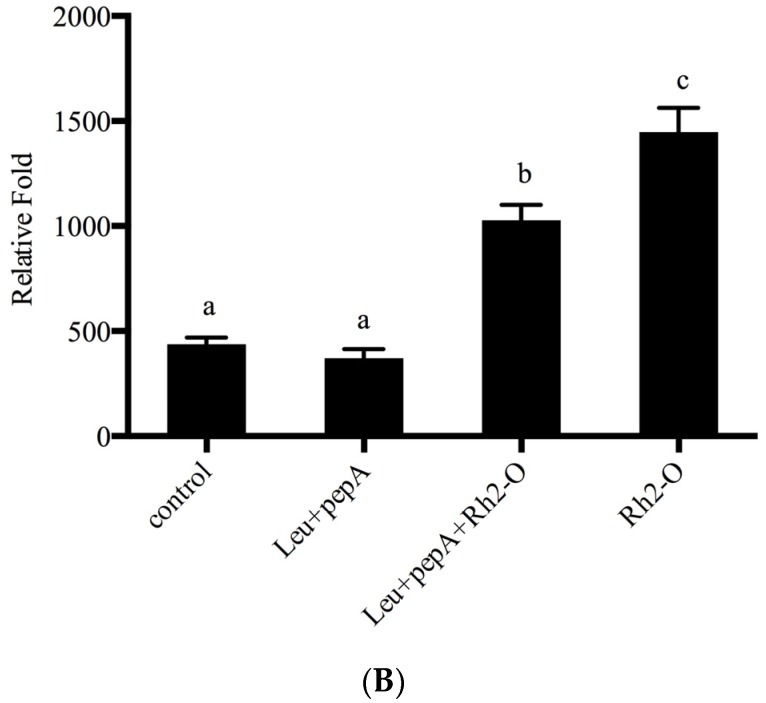
Effect of cathepsin inhibitor on the cleavage of Bid. (**A**) Western blot analysis for detecting tBid protein level after indicated treatment; (**B**) Histogram represents quantification of tBid protein expression levels in Rh2-O-stimulated HepG2 cell samples using Image J64 software (level of control cells/β-actin defined as 1). Results are presented as mean ± SD of three separate experiments. Bars with different letters are significantly different from each other (*p* < 0.05).

Previous studies have shown that lysosomes were involved in an apoptosis pathway through the cathepsin-tBid-caspase 3 pathway [24,25]. To assess whether cathepsins were involved in Rh2-O-induced apoptosis partially through regulating cleavage of Bid, the effects of Leu and pepA on Rh2-O-induced cleavage of Bid were examined. The results showed that Rh2-O induced Bid cleavage, and inhibitors partially reduced Rh2-O-induced tBid production (Figure 4), indicating that cathepsins mediated Rh2-O-induced cleavage of Bid.

### 3.5. The Release of Lysosomal Proteases Was Upstream of Mitochondrial Membrane Permeabilization

**Figure 5:**
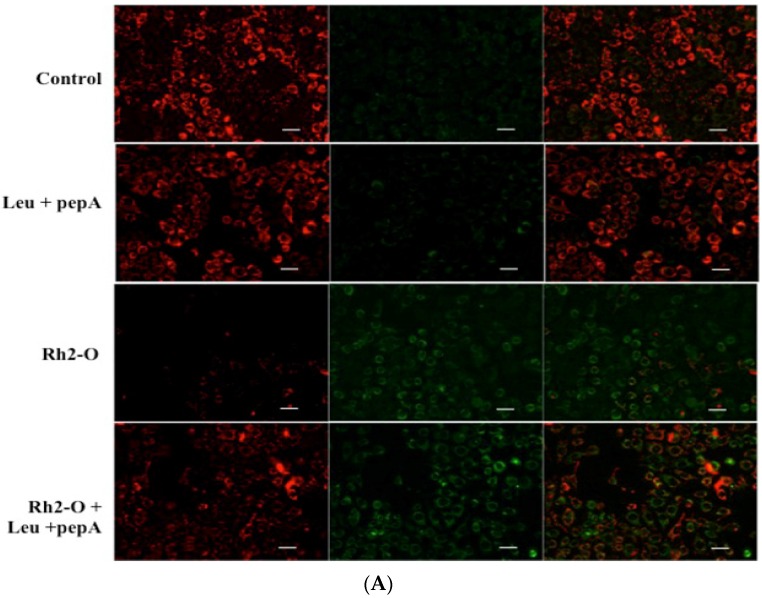

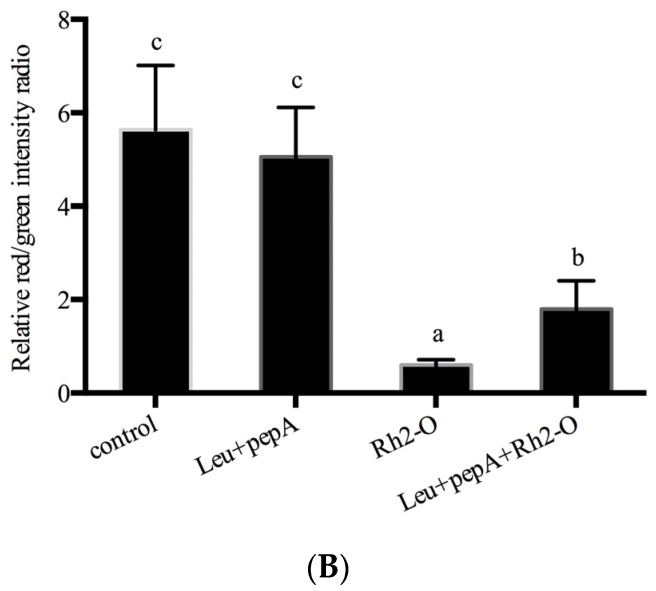
The decline of mitochondrial membrane potential (Δφm) stimulated by Rh2-O exposure in HepG2 cells. (**A**) After Rh2-O treatment with or without cathepsin inhibitor for 24 h, the levels of Δφm were detected by fluorescence microscope. The scale bar is 20 μm; (**B**) The relative red/green fluorescence intensity ratio in HepG2 cells after different treatments. Results are presented as mean ± SD of three separate experiments. Bars with different letters are significantly different from each other (*p* < 0.05).

It has been reported that lysosomes and lysosomal proteases might act as the amplifiers of apoptotic pathways which trigger the mitochondrial pathway (extrinsic pathway) accompanied by Δφm depolarization and the release of mitochondrion-associated pro-apoptotic proteins [26]. To assess whether cathepsins were able to affect the stability of mitochondrial membrane, potential changes were determined by JC-1 fluorescent staining. JC-1 color altered from red fluorescence to green fluorescence when the Δφm decreased. Changes of fluorescence in Rh2-O-treated cells were shown in Figure 5A,B after being pretreated with or without Leu and pepA. Compared to control cells, treatment with Rh2-O led to a marked reduction of red fluorescence and an increase of green fluorescence (*p* < 0.05). However, pretreatment with Leu and pepA significantly prevented mitochondrial membrane permeabilization (*p* < 0.05).

### 3.6. Bax and DRAM1 Involved in Lysosomal Membrane Permeabilization

**Figure 6:**
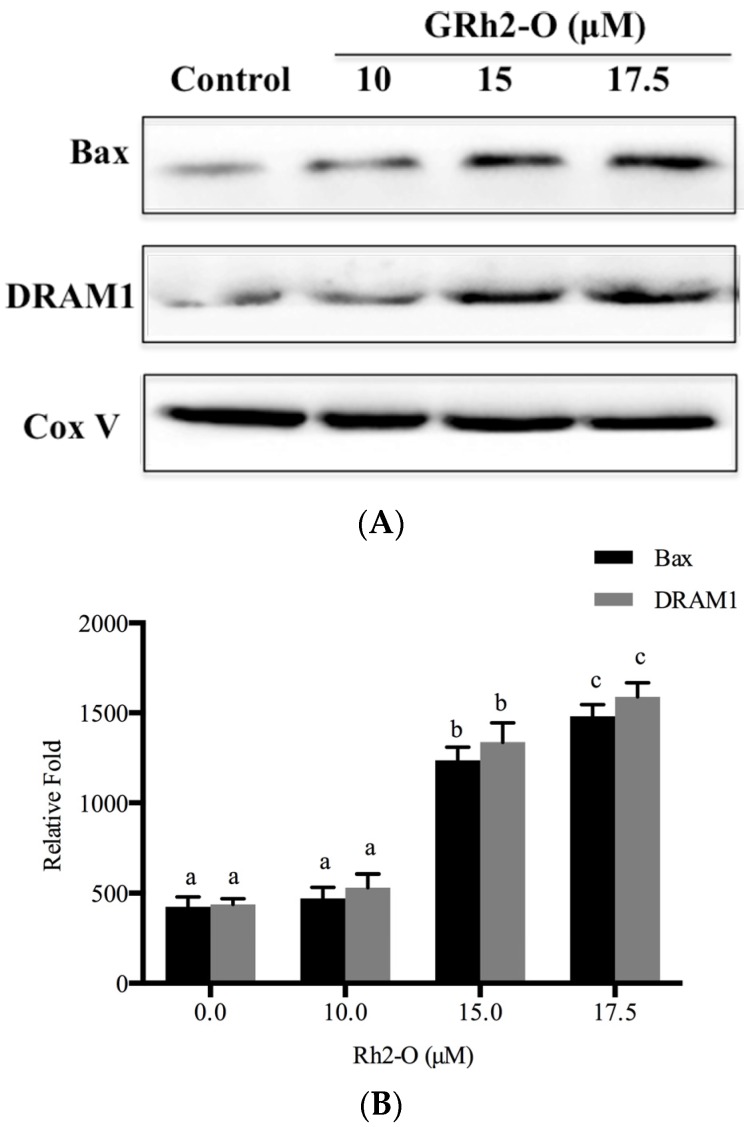
Bax and DRAM1 involved in lysosomal membrane permeabilization in Rh2-O-treated HepG2 cells (**A**) Western blot analysis for detecting Bax and DRAM1 protein levels on lysosomal membrane after indicated treatment; (**B**) Histogram represents quantification of lysosomal Bax and DRAM1 protein expression levels in Rh2-O-stimulated HepG2 cell samples using Image J64 software (level of control cells/β-actin defined as 1). Results are presented as mean ± SD of three separate experiments. Bars with different letters in each group are significantly different from each other (*p* < 0.05).

**Figure 7:**
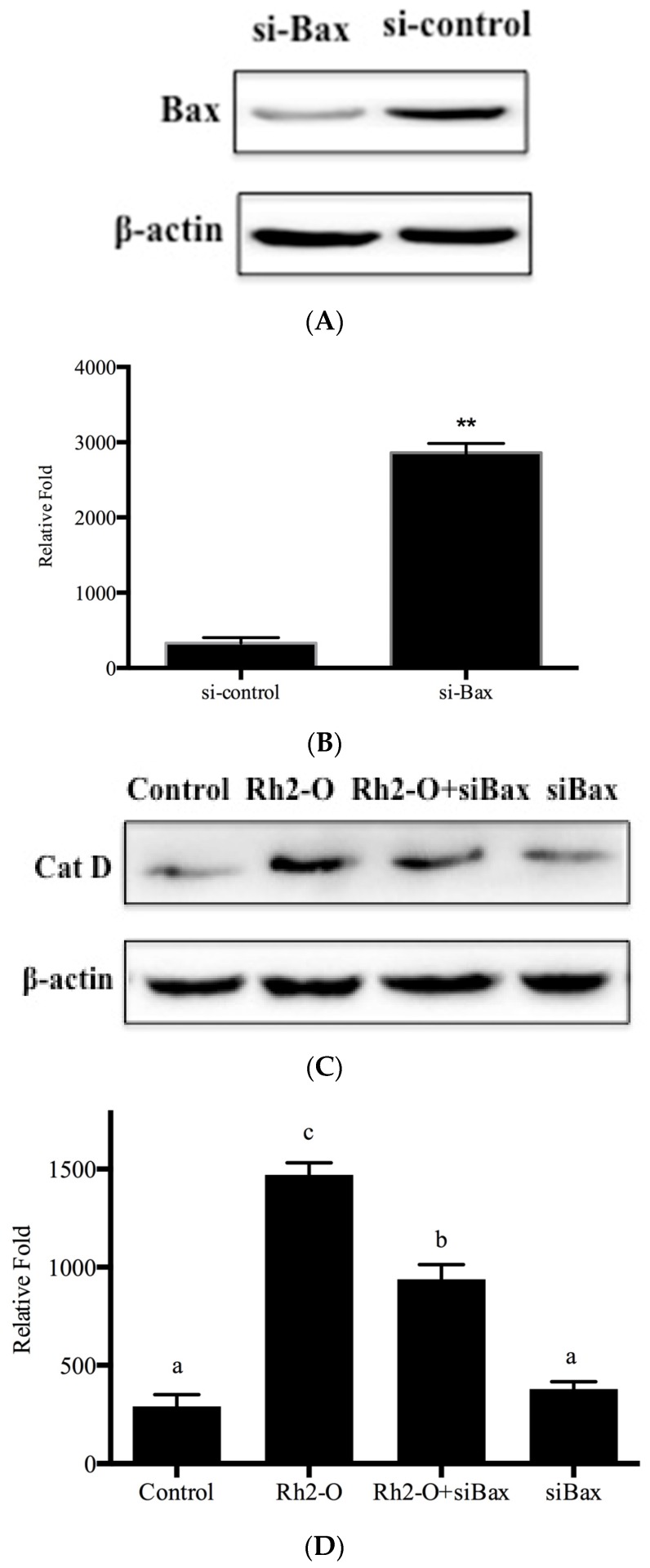
Effect of siBax on the release of Cat D. (**A**) Western blot analysis for determining Bax levels in HepG2 cells with or without knockdown of Bax; (**B**) Histogram represents quantification of Bax protein levels in HepG2 cells with or without knockdown of Bax. Results are presented as mean ± SD of three separate experiments. ** *p* < 0.005 *vs.* si-control group; (**C**) Western blots analysis for determining Cat D protein levels in cytosol after indicated treatment; (**D**) Histogram represents quantification of Cat D protein levels in Rh2-O-stimulated HepG2 cell samples with or without knockdown of Bax using Image J64 software (level of control cells/β-actin defined as 1). Results are presented as mean ± SD of three separate experiments. Bars with different letters are significantly different from each other (*p* < 0.05).

It was reported that Bax contributed to lysosomal destabilization by interaction with DRAM1, a lysosomal membrane protein [19]. To determine whether DRAM1 and Bax were involved in the regulation of Rh2-O-induced lysosomal membrane permeabilization, the protein levels of DRAM1 and Bax on lysosomal membrane were investigated in HepG2 cells. It is found that Rh2-O induced a significant increase in the protein levels of DRAM1 and Bax on lysosomal membrane (*p* < 0.05) (Figure 6). To test if Bax had a role in the release of cathepsins from lysosomes to cytosol, Bax was knocked down with siRNA (Figure 7A,B). As shown in Figure 7, silence of Bax inhibited Rh2-O-induced release of Cat D to cytosol. These results indicated that Bax could mediate the permeabilization of lysosomal membranes and induce the release of cathepsins.

## 4. Discussion

The principal findings of this study have demonstrated that lysosomes were involved in Rh2-O cytotoxic signaling. The results have shown that, during exposure of HepG2 cell line to Rh2-O , (1) lysosomal membrane permeabilization occurred in Rh2-O-induced HepG2 cell apoptosis; (2) lysosomal cathepsin inhibitors were associated with a reduction in cellular apoptosis; (3) the release of lysosomal cathepsins was the upstream of mitochondrial membrane permeabilization; (4) Bax was the upstream of lysosomal membrane permeabilization; (5) redistribution of lysosomal cathepsins into the cytosol was blocked by the inhibition of Bax. These results strongly support a regulatory pathway of Bax-lysosomes-mitochondria in Rh2-O-induced HepG2 cell apoptosis.

Ginsenoside Rh2 had restricted applications in food and medicine because of its poor intestinal absorption after oral administration [27]. In our previous study, Rh2-O was found to possess better absorption and anticancer activity *in vitro* [7]. Furthermore, we showed that Rh2-O induced caspase-dependent apoptosis via the intrinsic pathway [7]. Our current data extended these observations by defining the mechanisms of lysosomal membrane permeabilization induced by Rh2-O. Consistent with previous observations [28], this study showed that Rh2-O induced lysosomal membrane permeabilization with the release of Cat B and Cat D to the cytosol. To further determine whether cytosolic location of Cat B and Cat D were important for the pro-apoptotic action, HepG2 cells were pretreated with specific inhibitors for Cat B (Leu) and Cat D (pepA). The results showed that Leu and pepA significantly reduced the apoptosis induced by Rh2-O.

Accumulating evidence suggested that lysosomal enzymes might induce apoptosis either directly or indirectly, through proteolytic activation of procaspase or acting on the mitochondria to release pro-apoptotic factors [29]. Researchers found that there were three Cat D-specific cleavage sites in pro-apoptotic protein Bid, and these truncated Bid fragments cleaved by Cat D could activate the mitochondrial apoptotic pathway [30]. It is well known that tBid could translocate to the mitochondria to induce mitochondrial membrane permeabilization. Other investigators found a bidirectional-inverted relationship between the expression of Cat B and Apaf-1, the main component of the apoptosome [31]. These findings indicated that the lysosomal protease cathepsins could activate the lysosome-mitochondria apoptotic pathway. The present study thus examined the effects of lysosomal protease cathepsins on cleavage of Bid and mitochondrial membrane permeabilization. We found that the Cat B inhibitor (leu) and Cat D inhibitor (pepA) could inhibit Rh2-O-induced tBid production and Δφm depolarization, suggesting that Cat B and Cat D might be involved in the cleavage of Bid and mitochondrial membrane permeabilizationin in Rh2-O-treated HepG2 cells. Our results also indicated that lysosomal permeabilization occurred upstream of mitochondria dysfunction.

Bax, a pro-apoptotic member of the Bcl-2 family, has been long known to control mitochondrial membrane permeabilization during apoptosis [17]. Recently, Guan *et al* reported that Bax could be distributed to lysosomes to affect lysosome permeability and cathepsin release though interaction with DRAM1 [19]. In the present study, the results showed that Rh2-O induced a significant increase in the protein levels of DRAM1 and Bax in HepG2 cells. To examine the effects of Bax on lysosomal protease translocation, siRNA was used to knock down Bax. Knockdown of Bax partially inhibited Rh2-O-induced Cat D release from lysosomes. Altogether, it was possible that Bax was upstream of cathepsin release and essential for lysosomal membrane permeabilization during Rh2-O-induced apoptosis.

## 5. Conclusions

In conclusion, we have further characterized the cellular mechanisms underlying Rh2-O-induced HepG2 cell apoptosis. We discovered that Rh2-O was able to induce Bax to translocate to lysosomes, leading to lysosomal membrane rupture by interaction with DRAM1, and then lysosomal protease cathepsins relocated to the cytosol with subsequent Bid activation and mitochondrial membrane permeabilization. However, necrosis was also induced by Rh2-O in HepG2 cells, and this issue will be analyzed in detail in the course of a following study. Rh2-O might act as a potential anticancer compound for medical use. Further *in vivo* and clinical research is still needed.
